# CCL2: An Important Mediator Between Tumor Cells and Host Cells in Tumor Microenvironment

**DOI:** 10.3389/fonc.2021.722916

**Published:** 2021-07-27

**Authors:** Jiakang Jin, Jinti Lin, Ankai Xu, Jianan Lou, Chao Qian, Xiumao Li, Yitian Wang, Wei Yu, Huimin Tao

**Affiliations:** ^1^Department of Orthopedics, 2nd Affiliated Hospital, School of Medicine, Zhejiang University, Hangzhou, China; ^2^Orthopedics Research Institute of Zhejiang University, Hangzhou, China

**Keywords:** chemokines, chemokine receptors, inflammation, stroma, tumor microenvironment

## Abstract

Tumor microenvironment (TME) formation is a major cause of immunosuppression. The TME consists of a considerable number of macrophages and stromal cells that have been identified in multiple tumor types. CCL2 is the strongest chemoattractant involved in macrophage recruitment and a powerful initiator of inflammation. Evidence indicates that CCL2 can attract other host cells in the TME and direct their differentiation in cooperation with other cytokines. Overall, CCL2 has an unfavorable effect on prognosis in tumor patients because of the accumulation of immunosuppressive cell subtypes. However, there is also evidence demonstrating that CCL2 enhances the anti-tumor capability of specific cell types such as inflammatory monocytes and neutrophils. The inflammation state of the tumor seems to have a bi-lateral role in tumor progression. Here, we review works focusing on the interactions between cancer cells and host cells, and on the biological role of CCL2 in these processes.

## Background

Many immune cell subtypes, including myeloid-derived monocytes and macrophages, neutrophils, and T cells, are found to be considerably abundant in the tumor microenvironment (TME). Tumor-infiltrating cells are considered to favor tumor progression and immunosuppression, hampering the anti-cancer immune response.

The mechanisms of immune resistance and escape are complex and vary among different tumor types. Recently, the role of the chemokine–chemokine receptor axis in tumor progression has attracted interest (1). CCL2, also known as monocytic chemotactic protein 1 (MCP-1), was one of the first chemokines to be discovered and was found to possess strong chemotactic capability to recruit monocytes and macrophages. There is abundant evidence that overexpression of CCL2 promotes tumor metastasis, invasion, and immune resistance; however, some findings indicate that CCL2 expression can also initiate infiltration of anti-tumor inflammatory monocytes (1). The diverse mechanisms by which CCL2 regulates the tumor immune microenvironment are precisely regulated by tumor cells, tumor-infiltrating immune cells, and the tumor stroma. Thus, despite a considerable amount of work on the role of CCL2 in tumors, there is still no clear understanding or global consensus.

For this review, we collected studies from recent years focusing on CCL2 in the TME. These studies have enormously enriched our understanding of the origins and targets of this mysterious chemokine. This review is intended to summarize their findings and clarify the functions and regulation of CCL2 in tumors of different stage and different immunogenic status.

## Regulation of CCL2 Production

**Figure 1:**
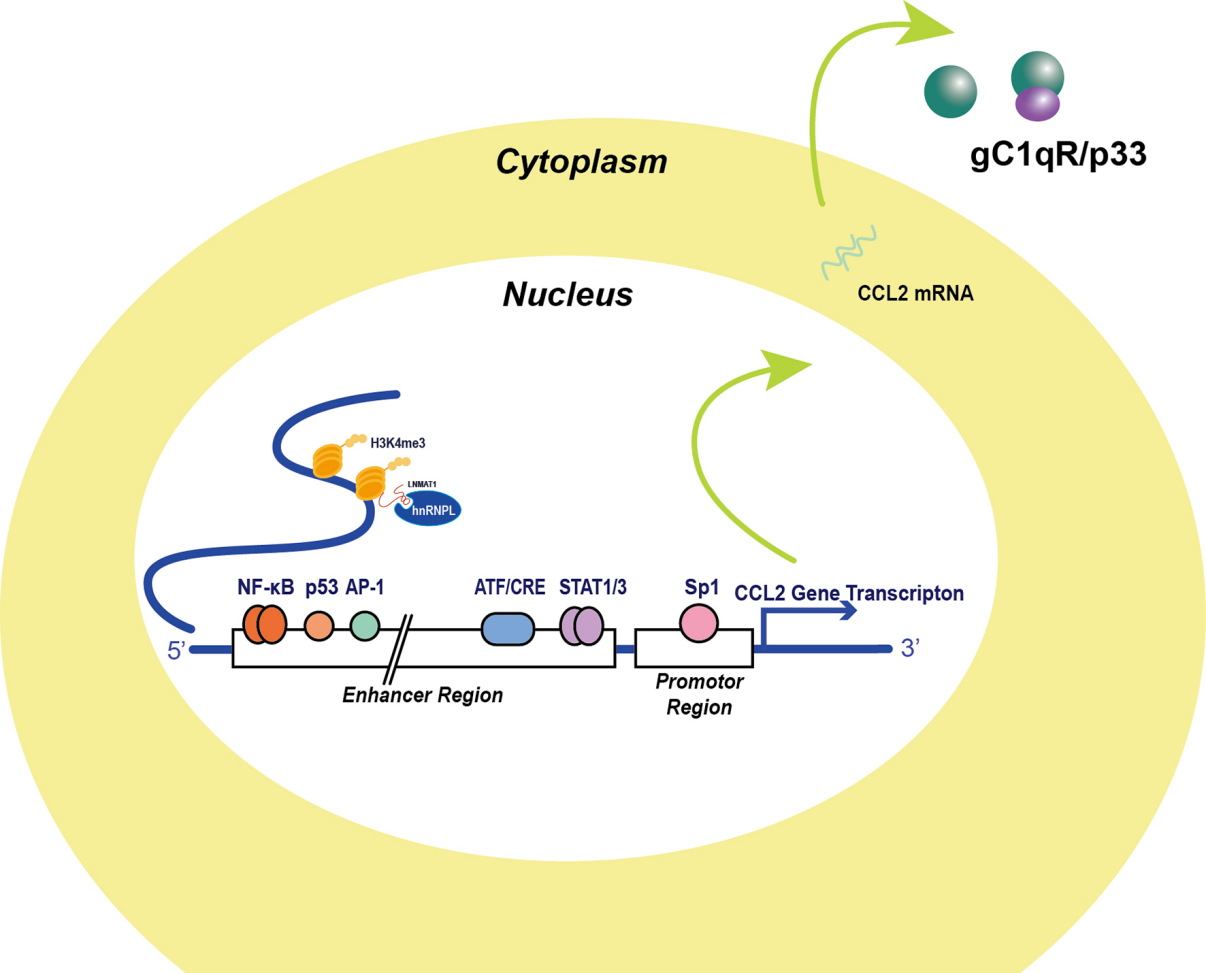
Transcription factors and post-translational factors in the regulation of CCL2 expression. Several transcription factors binding to CCL2 regulating sequences were identified in the 5’ UTR. The classical NF-κB signaling molecule RelA binding site locates approximately from position -2650 to position -2600 and Sp-1 binds to the promoter of the CCL2 gene, which are major activators of CCL2 expression. AP-1 was thought to be another major promotor of CCL2 transcription, but AP-1 binding activity appears irrelevant to CCL2 transcription activity. Additionally, p53 molecule binding to the enhancer sequence in cooperation with NF-κB signaling activation and stabilize NF-κB signaling. Apart from this, signal transducer and activator of transcription 1 and 3 dimers, which are also significant inflammatory signal molecules, transcriptionally prompt CCL2 expression. Moreover, Stress stimuli-induced factor ATF3 also binds to three ATF/CRE sites in the enhancer region and attenuates CCL2 transcription (2). Non-coding RNA like LNMAT1 recruits hnRNPL for H3K4me3 and enhances the CCL2 transcription (3).

CCL2 is synthesized and secreted mainly by monocytic cells. These have been the most commonly researched cell type in this context in recent years. Tumor cells have also been found to express CCL2, and multiple transcription factors overexpressed by cancer cell have been identified to affect CCL2 transcription (**Figure 1**).

Two NF-κB binding sites in the CCL2 gene in the distal enhancer region were successfully identified by Ueda in 1994 (4). The proximal GC box located at positions −64 and −59 was thought to be responsible for basal CCL2 gene expression, whereas the distal NF-κB binding site located between −2612 and −2603 was required for stimulation and enhanced cytokine production. Typical stimulations leading to CCL2 expression include TNFα, IL-1β, and phorbol-12-Myristate-13-Acetate (PMA) treatment. Tumor antigens can reprogram inflammatory cells and promote tumor immune invasion *via* NF-κB-induced CCL2 production. CCL2 production has been reported to be more inducible in HER2+/ER− breast carcinoma cells compared with HER2+/ER+ cells under EGF/HRG stimulation, and enhanced NF-κB transcription levels were detected in HER2+/ER− breast carcinoma cells (5). Emerging evidence indicates that the JAK/STAT pathway can also promote CCL2 production under stimulation (6–13). Neddylation is a reversible protein modification process mediated by NEDD8. Activated neddylation was involved in carcinogenesis, which indicated that neddylation could be a potential target for cancer therapy (14). Zhou et al. found that the neddylation process mediated by NEDD8 was positively correlated with CCL2 expression in lung cancer. Inhibition of the neddylation process significantly reduced levels of cancer-associated macrophages and tumor growth (15).

In recent years, many non-canonical factors have been reported to directly bind to the cis-element of the CCL2 gene or protein independent of classical pathways. Anders et al. (16) found that the globular C1q receptor (p33), a binding partner of C1q in the complement system, could stabilize CCL2 protein. *In vitro* studies have revealed high-affinity binding between CCL2 protein and p33, and recombinant p33 greatly enhanced CCL2 persistence and the migration capacity of THP-1 monocytes. Choi et al. identified NFAT5 as an independent factor that promotes CCL2 secretion in rheumatoid arthritis, leading to apoptosis resistance of macrophages (17). Lennard Richard et al. detected binding of Fli-1, a member of the Ets transcription factor family, to an Ets binding site within the CCL2 promoter, which activated gene expression. They also found that Fli-1 may work synergistically with NF-κB signaling to promote CCL2 gene transcription (18). Hacke et al. proposed a p53 binding site 2.5 kb upstream of the start site of the CCL2 gene, based on in silico analysis. This finding was confirmed *in vitro* by electrophoretic mobility shift assay and *in vivo* by ChIP assay (19). In summary, studies have unveiled several non-canonical pathways of CCL2 gene regulation that may contribute to pro-inflammatory responses.

## Non-Coding RNAs Involved in CCL2 Regulation

Non-coding RNAs have attracted considerable attention owing to their roles in regulating tumor behavior and have been shown to be closely correlated with CCL2 production and TAM reprogramming. Tumor-derived microRNA-375 was found to mediate tumor cell–macrophage interplay *via* CCL2; specifically, microRNA-375 could promote CCL2 production in tumor cells and induce macrophage migration (20). Hsu et al. studied miR-122, an anti-inflammatory microRNA, and found that its inhibition induced activation of RelB and subsequent release of pro-inflammatory chemokines, including CCL2, by macrophages (21). Long non-coding RNA LNMAT1 was shown to epigenetically activate the CCL2 gene by attracting hnRNPL to the CCL2 promoter, leading to histone trimethylation (22). Decreased CCL2 levels and macrophage infiltration density were observed in the LNMAT1-silenced TME. Circular RNA hsa_circ_0110102 functions as a molecular sponge to inhibit CCL2 transcription in hepatocellular carcinoma (HCC) cells, which further inhibits macrophage recruitment. Downregulation of hsa_circ_0110102 is closely related to poor prognosis in HCC patients. Thus, non-coding RNAs, owing to their functions as additional regulators in the TME, are emerging as potential therapeutic targets and as a focus of anti-cancer drug investigations (23). Other non-coding RNAs modulating CCL2 expression are listed in **Table 1**.

**Table 1:** Non-coding RNAs and mechanisms involved in CCL2 regulation.

Name	Cancer Type	Target	Up- or down-regulation of CCL2	Ref.
miR-339-5p	OSCC	TSPAN15	Down	(24)
miR-122	HCC	–	Down	(25)
MiR-124	OC	–	Down	(26)
miR-1246	BC	PRKAR1A, PPP2CB	Up	(27)
miR-1, miR-206, miR-31	LC	OXO3a/VEGF/CCL2	Up	(28)
miR-122	HCC	C/EBPα	Down	(29)
miR-511	AML	cyclin D1	Up	(30)
MiR-19a	GC	IκB−α	Up	(31)
miR-126/miR-126(*)	BC	SDF-1α	Down	(32)
miR-16	BC	IKKα	Down	(33)
miR-200c	BC	p65/RelA, JNK2	Up	(34)
LncHOTAIR	CRC	miR-206/CCL2	Up	(35)
Antisense IL-7	–	NF-κB/MAPK	Up	(36)
LncHOTAIR	HCC	–	Up	(37)

## The Biological Role of CCL2 in the TME

The TME has a supportive role in tumor progression and immune suppression (38). Myeloid cells and stromal cells have been found to abundantly accumulate in the TME (39, 40). The mechanism of immunosuppression varies; in recent years, chemokines and chemokine receptors have been found to have strong correlations with cancer-associated chronic inflammation and were thought to be responsible for the recruitment of immune cells. Emerging results indicate a considerable concentration of CCL2 in tumor tissue, suggesting that the presence of CCL2 may contribute to tumor spreading and pre-metastatic niche formation (**Table 2**). Here, we review several studies focusing on the effects of CCL2 on different host cells in the TME.

**Table 2:** The significance of CCL2 in tumor metastasis.

Origin of CCL2	Cancer type	Signaling involved	Mechanisms of metastasis	Ref.
Tumor cells	ESCC	NK-kB	regulatory T cells recruitment	(41)
Mesenchymal stem cells	GC	Wnt/b-catenin	GC-MSCs recruitment	(42)
CAFs	ESCC	TGFβ2-ERK	Paracrine capacity and pro-metastatic ability of fibroblasts	(43)
CAAs	Ovarian cancer	PI3K/AKT/mTOR	CCL2-CCR2 axis favorable for omental metastasis	(44)
TAMs	TNBC	Activation of the p-Src and p-Erk1/2 signaling	Enhanced proteolytic effect and invasion	(45)
Tumor cells	HCC	MYC/Twist1 signaling	Polarization of TAMs	(46)
Periodontal inflammation	BC	IL-1β/CCL2	Recruitment of TAMs and MDSCs	(47)
CAFs	BC	Activation of p-FAK and p-STAT3	Epithelial-mesenchymal transition	(48)
Tumor cells	LUAD	KLF6-SV1/Twist1/CCL2	Epithelial-mesenchymal transition; polarization of M2 macrophages	(49)
Tumor cells	IBC	IL8/STAT3	Epithelial-to-mesenchymal transition; Cancer-stem-cell-like and mesenchymal phenotype; M2 TAMs derived IL-8 secretion	(50)
Tumor cells	Double-Negative Prostate Cancer	PRC1/CCL2	Recruitment of M2 TAMs and Tregs	(51)
Tumor cells	GBC	PLEK/EGFR/CCL2	Epithelial-mesenchymal transition	(52)
Tumor cells	LLC	CCL2-pulmonary endothelial CCR2	Increased endothelial cells retraction and vascular permeability	(53)
CAFs	ESCC	TGFβ1/SMAD3	Increased metabolites from CAFs favorable for tumor progression	(54)
Lung residential macrophages	–	Tumor released micro-particles	Recruitment of inflammatory monocytes; Polarization of macrophages; Fibrin production and deposition.	(55)
Tumor cells	LUSC	Tumor cells-inflammatory monocytes interaction	Recruitment of inflammatory monocytes (IMs) expressing Factor XIIIA; Scaffold formation by IMs.	(56)
Interstitial macrophages	HCC	5-LOX/LTB4,CCL2-CCR2	Recruitment of CCR2+ alveolar macrophages	(57)
Tumor cells & myeloid cells	BC	Wnt1/E-cadherin	Recruitment of CD206/Tie2+ macrophage; Epithelial-mesenchymal transition;	(58)
Tumor cells	Bladder cancer	LNMAT1/CCL2	VEGF-C secretion; Recruitment of TAMs;	(3)
Tumor cells	BC	CCL2/IL-1β/ γδτ	γδτ cell derived IL-17; induction of immunosuppressive neutrophils.	(59)

## Tumor-Infiltrating Myeloid Lineage Cells

### Tumor-Associated Macrophages

Macrophages have long been considered to form the major component of the TME of many tumors. Evidence from clinical samples and *in vitro* studies illustrates the contributions of TAMs to tumor progression, invasion, metastasis, and immunosuppression (60). Wei et al. showed that crosstalk between tumor cells and TAMs promotes cancer metastasis, which could be hampered by blocking of CCL2 (20). There is also evidence that high levels of CCL2 promote polarization of TAMs to an immune-regulatory phenotype, which is considered a consequence of tumor cell-mediated immune modulation. Interactions between tumor cells and macrophages often promote the immunoregulatory function of the host immune cell, thereby facilitating tumor progression (61–63).

#### Influence of CCL2 on TAMs in Cooperation With Multiple Signaling Molecules

Interactions of signaling molecules and signal transduction in the TME dynamically regulate tumor behavior and reprogramming of TAMs. CCL2 may enhance the phagocytotic ability of tumor-entrained macrophages and initiate the subsequent release of cell-death-related molecules such as RANTES, MIF, CXCL-12, and IFNγ (64). The process is activated by platelet-derived microparticles; however, macrophages of this subtype may also promote epithelial-to-mesenchymal transition, making the tumor more prone to metastasis.

IL-1β is a classic pro-inflammatory cytokine secreted by TAMs, which has been found to induce immunosuppression in many tumor types. IL-1β-deficient tumor-bearing mice were shown to have lower levels of CCL2 in tumor tissue, which resulted in decreased macrophage infiltration and CD11b+ dendritic cell maturation (65). Conversely, CCL2 was reported to induce IL-1β expression in TAMs (59). The regulation mechanism of IL-1β remains unclear. Further, CCL2 could not activate IL-17+ γδ T cells and neutrophils in the absence of CCR2+ TAMs, indicating that TAMs are important mediators and targets of CCL2 function.

GM-CSF is a key cytokine responsible for differentiation of M2 TAMs. Yoshimura et al. identified GM-CSF derived from 4T1 cells as an important inducer of CCL2 expression in macrophages (66). They also recently reported that GM-CSF-deficient tumor cells did not show retarded growth rates and had no effect on overall CCL2 expression levels in 4T1 tumor tissue, indicating that non-myeloid-cell-derived CCL2 is also influenced by GM-CSF deletion (21).

#### Metabolic Dysfunction of Tumor Induces CCL2 Expression and TAM Recruitment

Tumor metabolism is frequently observed to be abnormal in tumor cells and in non-tumor cells in the TME. Mutations of isocitrate dehydrogenases 1 and 2 (IDH1/2) have been identified as specific biomarkers of brain tumors (22). Moreover, 2-hydroxyglutarate, the major metabolite of IDH1/2, could initiate activation of pro-inflammatory microglia, a specific subtype of macrophages in brain tissue. Pretreatment of primary microglia with glioma-cell-conditioned media significantly suppressed NF-κB activity and subsequent CCL2 gene expression (23). Anisiewicz et al. demonstrated the impact of calcitriol, the key metabolite of vitamin D metabolism, on metastatic mammary gland cancer (67). Administration of vitamin D compounds increased plasma CCL2 and Arg1 expression in tumors, contributing to increased macrophage infiltration in the TME.

Mitochondrial dynamics have an important function in the tumor immune response. Mitochondrial dysfunction is often observed in various tumor types (68). Dynamin-related protein 1 (Drp1), a protein responsible for mitochondrial fission and fusion dynamic balance, was found to regulate mitochondrial DNA (mtDNA) stress (69). Whereas cytosolic mtDNA stress was reported to significantly abrogate oxidative phosphorylation and trigger the calcium-dependent adaptive immune response (70), Drp1 overexpression-induced mitochondrial fission promoted CCL2 expression, resulting in CD163+ TAM infiltration in HCC. Similar results were obtained by Grasso et al, who found that mtDNA deprivation of 4T1 cells greatly hampered tumor formation ability and decreased CCL2 gene expression (70).

#### Oncogenic Mutations Contribute to Immune-Suppressive TAM Recruitment by Inducing CCL2 Expression

Increasing evidence indicates that oncogenic mutations participate in tumor angiogenesis and formation of an immunosuppressive microenvironment. Kazantseva et al. examined the relationship between TP53 isoform Δ133p53β and CCL2 expression and their contributions to glioblastoma progression. Glioblastomas with high Δ133p53β expression exhibited an enhanced hypoxia state, more CCL2 expression, and increased infiltration of CD163+ TAMs (71).

Abnormal activation of epidermal growth factor (EGFR) signaling is often found in various cancer types. Correlation analysis by An et al. showed that EGFRvIII enrichment was commonly found with high CCL2 expression and positively correlated with macrophage infiltration (72). A clinical trial reported that erlotinib, an EGFR inhibitor approved by the Food and Drug Administration, decreased serum CCL2 levels in non-small-cell lung cancer (NSCLC) patients (73). Mutation of the retinoblastoma (RB) tumor suppressor gene enhances CCL2 production in multiple cell lines, resulting in the recruitment of TAMs, myeloid-derived suppressor cells (MDSCs), and regulatory T cells (Tregs).

RB mutation results in increased fatty acid oxidation, which can upregulate mitochondrial superoxide and activate JNK signals. This indicates that CCL2 plays an important part in oncogenic mutation-induced immune escape by regulating fatty acid metabolism. The crosstalk between oncogenic mutation and chemokine signals requires further investigation (74).

#### CCL2 Assists Tumor Metastasis *via* TAMs

CCL2-induced tumor spreading and metastasis are regarded as important results of TAMs recruitment and polarization (22).

In colorectal cancer, CCL2 was found to be strongly correlated with endothelial–mesenchymal transition, and inhibition of CCL2 significantly reduced macrophage infiltration and tumor metastasis mediated by circulating cancer cells (20). Macrophage-derived CCL2 can directly target non-neoplastic epithelial cells and transform them into more invasive ones. MCF10A cells receiving CCL2 signals show enhanced expression of ERO1-α and MMP9, which is crucial to the invasiveness of MCF10A cells co-cultured with TAMs (62).

Tissue-resident macrophages or myeloid-derived specific cells assist metastasis in many cancers. Skeletal metastasis also has been shown to be driven by CCL2 (23). There is evidence that CCL2 derived from osteoblasts is responsible for osteoclast recruitment (67). Synergic effects of parathyroid hormone derived from tumor cells and CCL2 derived from bone host cells contribute to bone remodeling and formation of a premetastatic environment (68). Interstitial macrophages overexpressing CCL2 are responsible for infiltration of CCR2-expressing alveolar macrophages from the blood stream, which facilitates lung metastasis (69).

Tumor-secreted microparticles participate in the modulation of tumor-infiltrating lymphocytes and myeloid-derived immune cells. Circulating tumor microparticles can reprogram TAMs; this is conducive to lung metastasis. Tumor-derived microparticles assist lung residential macrophages to take up most of the lung parenchyma and produce more CCL2 (70). Hypoxia-induced ejection of CCL2-rich exosomes from tumor cells enhances oxidative phosphorylation in macrophages and tumor progression (71). Neoadjuvant chemotherapy was shown to induce release of microvesicles from breast cancer cells containing more CCL2 molecules; it also enhanced infiltration of Ly6C+CCR2+ monocytes recruitment in the lung pre-metastatic site (72).

### Tumor-Associated Neutrophils

Tumor-associated neutrophils (TANs) in the TME function as both anti-tumor and pro-tumor regulators in tumors with various immunogenic characteristics (75, 76). The CD66b+, CD117+, CD11b+ neutrophil subsets are immature TANs. Neutrophils expressing CD66b, CD11b, PD-L1, and high levels of CD170 represent the pro-tumor subtype, whereas CD66+, CD11b+, CD170 low, and CD177+ neutrophils have an anti-tumor role (77, 78). The inflammatory state of the TME is significantly dependent on the activation state of TANs (79). TANs are recruited in response to CCL2 secreted by tumor cells and stromal cells in the tumor environment and may automatically secrete CCL2 to amplify the inflammation response.

#### Bilateral Roles of TANs in Tumor Progression

Neutrophils have diverse roles in altering the metastatic ability of cancer cells in response to different stimulations.

Investigations using multiple metastatic models have provided evidence that neutrophils entrained by CCL2-expressing tumor cells are capable of attenuating lung metastases (80). CCL2 secreted by tumor cells is critical for the killing capacity of neutrophils. CCL2 knockdown in primary tumor cells resulted in retarded tumor growth but earlier development of lung metastases. Enhanced TGFβ secreted by tumor cells was also found to weaken the killing capacity of tumor-entrained neutrophils and transform TANs into a pro-tumor phenotype (81). Tumor cells can also induce CCL2 expression in TANs in a paracrine manner, resulting in a protective effect against chemotherapy (82).

Circulating neutrophils may have the opposite role in tumor metastasis. In mammary tumors, CCL2 induces crosstalk between IL-1β-producing TAMs and γδ T cells, eventually results in immunosuppressive neutrophil entrainment to form a pre-metastatic niche (59). A high level of CCL2 in primary mammary tumor tissue is the major initiator of the tumor inflammation state *via* recruitment of inflammatory monocytes and release of IL-1β. IL-1β was also found to be responsible for circulating neutrophil polarization, which is dependent on IL-17 produced by activated γδ T cells. The interaction between TAMs and TANs also maintains the inflammatory balance in the TME. CCR2-deficient mice did not show reduced tumor inflammation, but MMP9+ neutrophils were found to be recruited in the TME (83).

#### Cytokine-Dependent Regulation of the Killing Capacity of TANs

CCL2-mediated CCR2+ monocyte recruitment in the tumor was shown to significantly enhance the killing capacity of neutrophils (84). Inflammatory monocytes in the TME release high levels of IFNγ, resulting in activation of killer neutrophils (TMEM173+ neutrophils). Yoshimura and Takahashi also identified IFNγ as the critical cytokine for neutrophil survival and the production of CCL2 by neutrophils (1, 85). Moreover, HCC-secreted CCL2 induced PD-L1 expression in tumor-infiltrating neutrophils, which hampered T cell-dependent tumor immunity. TNF family members function as critical signals for neutrophil activation (86). In 1998, Chuluyan et al. confirmed the chemoattractant function of TNFα-treated neuroblastoma cell supernatant for neutrophils, in which CCL2 upregulation was detected (87). Further, Yamashiro et al. reported that activated monocytes could effectively stimulate CCL2 expression in neutrophils, and that this effect could be interrupted by anti-TNFα-IgG (88). The following year, they confirmed the indispensable role of TNFα in CCL2 production in neutrophils (89). Cancer-associated mesenchymal stem cells (CA-MSCs) interact with neutrophils and enhance their CCL2 and TNFα production. Reciprocally CA-MSCs are differentiated to cancer-associated fibroblasts (CAFs) assisted by TANs (90). A similar finding was reported by Cheng et al. in their research on HCC (13). IL-6-STAT3 signaling plays an important part in these crosstalk phenomena.

## Tumor-Associated Stromal Cells

The tumor stroma is abundant in the TME and functions as a physical barrier to exhaust the immune response against tumors (91).

### Cancer-Associated Fibroblasts

CAFs express multiple surface antigens and are considered to participate in intimate crosstalk with tumor cells and immune cells. In recent years, CAFs were identified as an important source of CCL2, suggesting that they may regulate tumor immunity in an inflammation-dependent manner.

#### Fibroblast Activation Protein Is Closely Associated With CCL2 Production in CAFs

FAP is the classical biomarker for CAFs. Evidence indicates that FAP maintains CCL2 production by CAFs *via* continuous JAK2–STAT3 signaling (92). The activation of CCL2 by FAP is probably due to STAT3 phosphorylation rather than modification of the CCL2 protein (93). Geng et al. investigated the anti-tumor effects of a FAPα-targeted tumor vaccine in a murine model of breast cancer and found evidence that the FAPα vaccine significantly enhanced CD8+ T cell infiltration. CCL2 expression decreased, leading to a decrease in the infiltration rate of MDSCs after vaccine administration (94). CAFs were also found to directly enhance tumor growth *via* the CCL2–CCR2 axis in breast carcinoma (95). CCR2+ cancer cell invasiveness was enhanced by fibroblast-derived CCL2 *via* SRC and PKC activation. Although the CCL2–CCR2 axis is primarily considered to be a chemokine pathway involved in myeloid cell-participated immune regulation, CCR2-expressing tumor cells can also directly interact with CCL2 for itself activation. The relationship between the tumor stroma and inflammation in the TME has aroused increasing interest in recent years.

The TGFβ family consists of classical signaling molecules that are responsible for tumor stroma formation. Xu et al. focused on the mechanism of TGFβ2 in promoting esophageal squamous cell carcinoma (ESCC) metastasis (43). Collectively, their results showed that TGFβ2 production and secretion had significant responsibility for tumor angiogenesis and that CAFs have an important role in this process. Moreover, CAF-derived CCL2 was one of the most probable factors for tumor angiogenesis. These findings were validated by *in vivo* studies. Dituri et al. reported that TGFβ1-treated HCC-derived fibroblasts showed inhibited CCL2 production (43). Patient-derived CAFs were subjected to single-cell RNA sequencing to identify classical CAF biomarkers and divided into six subpopulations based on their top differentially expressed genes. CAF-derived CCL2 was proved to promote myeloid cell recruitment and maintain a chronic inflammatory state in the TME (96).

#### Interaction Between CCL2+ CAFs and Myeloid Cells Assists Immune Escape

Several recent studies have focused on CAF-derived CCL2 in the TME and its role in regulating migration and maturation of myeloid-derived cells in various tumor types. MDSCs are believed to have an independent role (97). Xiang et al. found that the abundance of CAFs in lung squamous cell carcinoma were positively correlated with monocytic myeloid cell abundance (96). CAFs were able to recruit CCR2+ monocytic cells and polarize such cells into MDSCs, which hampered CD8+ T-cell-dependent tumor immunity. Early recruitment of macrophages is correlated with cancer initiation, which includes formation of a favorable TME. CAF recruitment and fibrosis stroma formation in tumors are also dependent on macrophage recruitment (98). The role of fibroblasts in the early progression of ductal carcinoma *in situ* remains to be explored. Brummer et al. identified the CCL2–CCR2 axis as a critical mediator of this process (99). CCR2 overexpression in mammary tumor cell line SUM225 significantly enhanced survival and migration capacity. Increased infiltration of CCL2+ fibroblasts was also observed.

### Cancer-Associated Adipocytes

Epidemiologic research has identified obesity and excessive fat accumulation as risk factors in tumor patients (100, 101). Accumulating evidence indicates that high levels of adipocyte accumulation in the TME may be supportive of tumor progression, metastasis, and immunosuppression (102). Adipokines are termed as a bunch of cytokines secreted by adipocytes, including leptin, adiponectin and CCL2. In recent years, abnormally increased level of various adipokines was often observed in various cancer types (103). Various adipokines are pro-inflammatory, which is activated by NF-κB signaling (104). CCL2, as an important adipokine, mediates crosstalk between cancer cell and adipocytes (105, 106).

Infiltration of adipose stromal cells into in tumor tissue enhance CCL2 production, contributing to more recruitment of myeloid-lineage cells (98). Uchiyama et al. found that intermittent hypoxia upregulated CCL2 expression in adipocytes (107). Interaction between cancer cells and adipocytes is essential for adipocyte polarization that favors tumor progression (108). In prostate cancer, tumor-cell-derived IL-1β could reprogram adipocytes to have a pro-inflammatory phenotype, as evidenced by upregulation of CCL2 and COX2 (109). The CCL2–CCR2 axis has an impact on adipocyte differentiation. CCR2 inhibition by cenicriviroc significantly reduced expression of proteins related to adipose differentiation in hepatocytes, which prevented hepatocyte steatosis and carcinogenesis (110, 111). It was shown that the CCL2–CCR2 axis linking cancer cells with omental adipocytes supported peritoneal metastasis of ovarian cancer (44). Data analysis also showed that the CCL2, which enhanced tumor migration ability *via* the PI3K/AKT/mTOR pathway, is originated from adipocytes, not the tumor cell.

### Mesenchymal Stem Cells

The tumor stroma has a supportive role in many tumor types; this is mainly mediated by cancer-associated MSCs (CA-MSCs) and cell subtypes differentiated from MSCs (112). TA-MSCs migrating to tumor regions are involved in multiple mechanisms. There exists a complicated chemokine network involving CA-MSCs and tumor cells (8, 50, 113–115). Canonical therapeutic approaches such as chemotherapy can induce MSC enrichment in the TME. TA-MSCs also have a bilateral role in the TME, indicating that such cell subtypes have high plasticity and could be tuned for anti-cancer therapy (116, 117).

Experimental analysis has demonstrated that MSCs show enhanced PD-L1, TGFβ, and CCL2 expression in tumors (118). Pasquier et al. reported that CCL2/CCL5 secreted by MSCs induces chemoresistance in ovarian cancer. CCL2 promoted PYK2 phosphorylation in ovarian cancer cells *via* IL-6 secretion in a coculture system.

Biomechanics stimulation has also proved essential for MSC differentiation by remodeling the tumor matrix (119). Wong et al. found that soft extracellular matrix remodeled mesenchymal stromal cells into an inflammatory phenotype by inducing monocytic CCL2 secretion (120). Forced changes in the tumor promote pro-invasive remodeling of tumor-associated mesenchymal stem-like cells *via* CCL2-mediated activation of myosin light chain 2 (121). Therapy-induced reaction oxygen species production was also found to be correlated with MSC activation. Application of photodynamic therapy to MSCs resulted in decreased secretion of CCL2, which attracted TAM infiltration (122).

## T Cells

The hampered anti-tumor response is characterized by exhaustion of cytotoxic tumor-infiltrated T cells and abnormal Treg activation (123). Emerging evidence indicates that the CCL2-CCR2 axis could influence T cell-mediated tumor immune response, which is complicated due to the variety of T cell subtypes (124–126).

### Cytotoxic T Cells

The early study revealed that CCL2-null 4T1 cells elicited enhanced cytotoxic T cell recruitment and IFN-γ secretion *in vivo* and *in vitro*, while CCL2-deletion did not show enhanced immunogenicity of the total tumor (127). Of note, the author pointed out that increased cytotoxic T cell function could not necessarily imply increased immunogenicity, which was also presented by Knight, D. A., et al. (128), but enhanced immunogenicity, such as immunogenic cell death, often results in cytotoxic T cell activation (129). CCL2 also could indirectly reinforce or frustrate cytotoxic T cells by myeloid-derived cells, like monocytic-derived dendritic cells, MDSCs, or TAMs (124, 125, 130, 131). NF-κB activation is commonly considered as a signal in favor of tumor cell survival in many cancers. However, NF-κB-induced CCL2 expression was reported to maintain T cell-mediated immune surveillance (132). The author also hypothesized that CCL2 expression level could predict a patient’s response to immunotherapy.

The anti-tumor ability of γδ T cells attracts great attention in immunotherapy. It was reported that the CCL2-CCR2 axis possesses chemotaxis property toward cytotoxic type 1 γδ T cells in the TME of B16 melanoma tumor model (133). Conversely, type 1 γδ T cells were also found to be immunosuppressive in other cancer types (134).

### Adoptive T-Cell Immunotherapy

Adoptive T-cell immunotherapy is an emerging therapeutic strategy attracting increasing attention in recent years. The early study elucidated the chemotaxis ability of CCL2 toward adoptively transferred T cells (135). Since CCL2 secreted by tumors could not become a chemoattractant towards CCR2^low^ CD8+ T cells, generation of the functional CCR2-expressed CAR-T cells were reported to better localize in the tumor microenvironment (136, 137). Enhanced tumor trafficking effect of CAR-T by expressing CCR2 could be a potential strategy in combination with other CAR-T modification approaches to improve CAR-T therapy.

### Regulatory T Cells

Regulatory T cell, which refers to CD4^+^CD25^+^Foxp3^+^ T cell, is the major T cell subtype in response to CCL2. Chang et al. first reported Treg recruitment in response to TAMs and microglia in glioblastoma multiforme (133). The CCL2–CCR2 axis has a role in the process of Treg recruitment (134). Resistance is commonly encountered in radiotherapy treatment of solid tumors in clinical settings. Enhanced CCL2 release by irradiated tumor cells contributes to the recruitment of inflammatory monocytes and CCR2+ Tregs. Loyher et al. also observed the dominant role of the CCL2–CCR2 axis in trafficking Tregs; furthermore, they found that preferential clearance of CCR2-positive Tregs by low-dose cyclophosphamide significantly improved the prognosis of pre-clinical models receiving immunotherapies (138). Liu et al. reported that zoledronic acid could inhibit the interaction of breast cancer cells with Tregs by downregulating cancer-cell-derived CCL2 (139). Li et al. found that RB inactivation not only promoted tumor proliferation but was also responsible for enhanced Treg recruitment *via* CCL2 (140). However, conventional T cells accepting tumor antigens show low CCR2 expression compared with Tregs, which indicates a specific function of tumor antigens on Tregs. CCL2 blockade was reported to significantly enhance vaccine-mediated cancer immune response (141). It was also reported that tumor-specific vaccination induced migration of CCR2+ Tregs into the tumor region. Che et al. reported that a therapeutic vaccine for cervical cancer resulted in decreased infiltration of Foxp3+ Tregs and downregulation of CCL2 (142).

Circulating Tregs are not the only T cell subset to express CCR2. Ge et al. found that bone marrow (BM)-resident Tregs, but not naïve T cells in the BM, could specifically express CCR2 under tumor antigen activation (143). These Tregs possess a strong migration capacity and can infiltrate into TME during cancer development together with the development of adaptive tumor immunity. CCL2 levels are not always positively correlated with abundance of infiltrating Tregs; this varies according to the tumor type and inflammatory state. Jia et al. reported that in NSCLC, administration of an EGFR tyrosine kinase inhibitor significantly reduced Treg infiltration, accompanied by enhanced CCL2 expression (144).

Cytotoxic T cell abundance and activation state are necessary parameters during research and clinical immunotherapy. Effective T cell infiltration and activation require multiple stimulations and are under complicated control. As discussed above, CCL2 expression in the TME elicits chemotaxis to circulating T cells and the CCL2-CCR2 axis contributes to the trafficking of T cells in the TME. CCL2 even directly regulates cytotoxic T cell activation. To summarize, the myth of CCL2 mediation in T cell behavior is mainly due to the complex T cell subtypes. It is necessary to validate the CCR2 expression in different T cell subtypes.

## Concluding Remarks

The immune response against tumors is compromised for many reasons, particularly chronic inflammation in the TME. By reviewing research on multiple tumor-infiltrating host cells, we have clarified the capability of CCL2 to initiate tumor inflammation. Pro-inflammatory stimulation and signaling molecules such as TNFα activate CCL2 transcriptionally, and oncogenes such as p53 and RB also directly regulate CCL2 expression. Several non-coding RNAs alter the inflammation state of the tumor through post-transcriptional effects on signaling pathways such as NF-κB and VEGF signaling.

**Figure 2:**
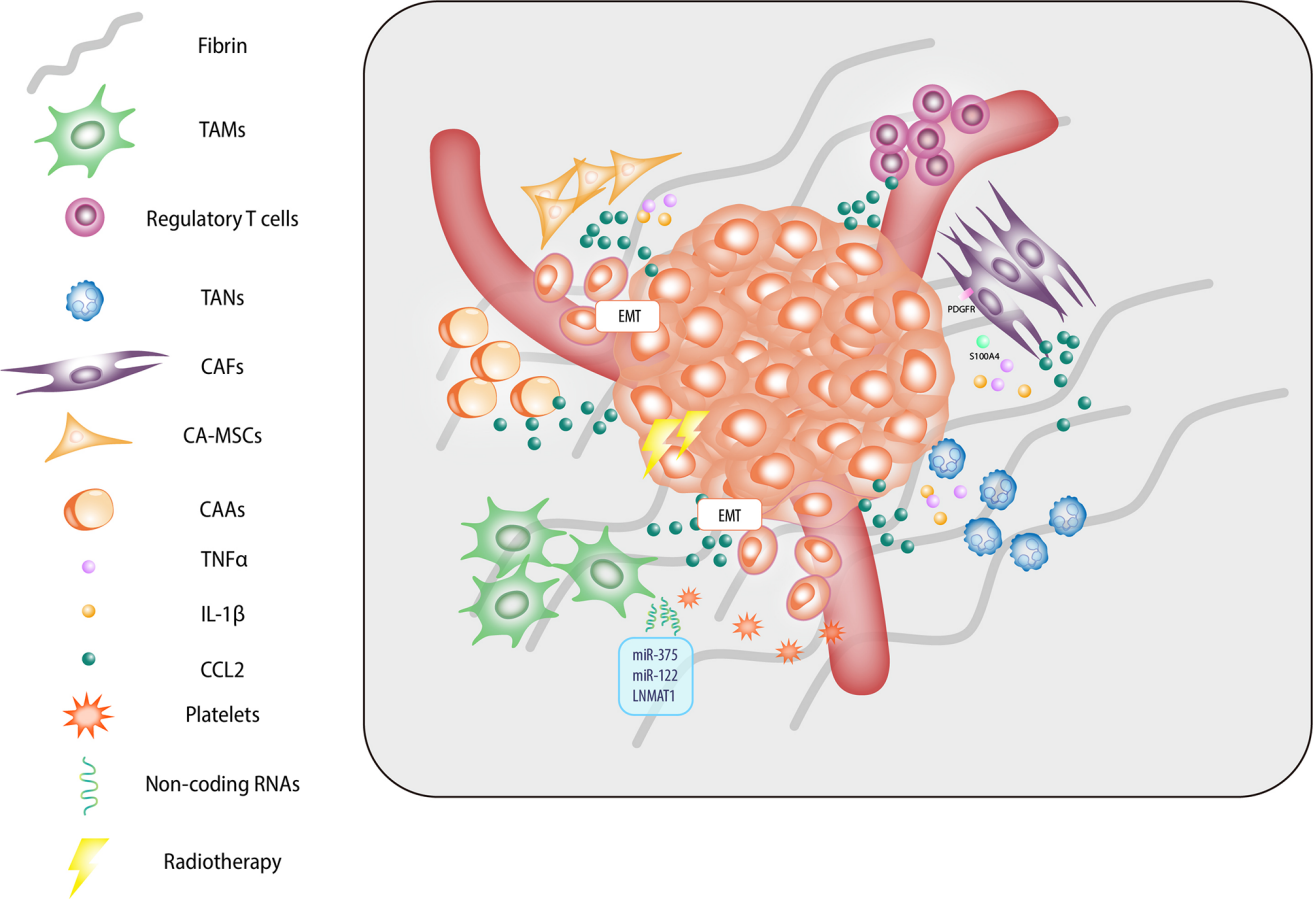
Host cells are involved in the tumor microenvironment.

Tumor cells possess the ability to actively secrete CCL2. Among the many reasons for this, genetic mutations and metabolic dysfunction have been most investigated. Classical chemotherapy and radiotherapy for cancer also stimulate CCL2-dependent chronic inflammation and tumor survival. Drug-induced fibrosis builds barriers against effective infiltration of tumor-antigen-presenting immune effector cells. CCL2 is responsible for the recruitment of immune effector cells, including macrophages, MDSCs, MSCs, and Tregs (**Figure 2**). Studies have shown that CCR2+ TAMs, CAFs, and cancer-associated adipocytes serve as additional sources of CCL2; these findings demonstrate the non-canonical roles of CCL2 in fibrosis generation, mesenchymal–epithelial transition, paracrine-induced tumor invasion, and angiogenesis.

Tumor-infiltrating macrophages, MSCs, and neutrophils have a high potential for plasticity, and their activation states are highly cytokine-dependent. IL-1β and TGF-β have crucial roles in CCL2-induced macrophage polarization and tumor-entrained neutrophil education, which weaken cytotoxic capacity and are also correlated with inflammation-induced immunosuppression. IFNγ and TNF-family cytokines have been reported to maintain the anti-tumor subtype of TANs. Other reviewers have also highlighted the bilateral role of chronic inflammation in cancer progression (145). Interestingly, increased IFNγ and TNF levels in tumors are considered to represent valid evidence of cytotoxic T cell activation in the TME, while these cytokines also function as stimulators of CCL2 production, which could simultaneously induce regulatory T cell trafficking and immune suppression. These seemingly contradictory phenomena imply that there exist more complicated mechanisms of CCL2 regulation. Further investigation of the underlying mechanisms could focus on the following aspects: (i). additional transcription factors that may be involved in CCL2 mRNA synthesis; (ii). potential post-transcriptional regulators of CCL2 mRNAs, such as circular RNAs; (iii). unexpected chemical modifications or protein interaction networks involving CCL2 molecules; and (iv). the CCL2-CCR2 axis functions diversely according to specific cell subtypes. The CCR2 functional researches on specific cell subtypes are required to better explain the CCL2 effect. To conclude, CCL2 has both anti-tumor and pro-tumor effects, depending on the interaction between cancer cells and host cells. To facilitate anti-tumor therapies, the duration and range of CCL2’s functions should be considered and modified where necessary.

## Author Contributions

JJ finished the collection of references and wrote this review article. WY and HT performed corrections to this article. JLo, JLi, AX, and Chao Qian gave precious suggestions on the design and content of this review. All authors reviewed and approved the final version of the manuscript.

## Funding

This study was supported by a grant from National Natural Science Foundation of China (No. 81872181, No. 82002836, No. 81702662), Natural Science Foundation of Zhejiang Province (No. LY20H160025, LY21H160034, and LY21H060003).

## Conflict of Interest

The authors declare that the research was conducted in the absence of any commercial or financial relationships that could be construed as a potential conflict of interest.

## Publisher’s Note

All claims expressed in this article are solely those of the authors and do not necessarily represent those of their affiliated organizations, or those of the publisher, the editors and the reviewers. Any product that may be evaluated in this article, or claim that may be made by its manufacturer, is not guaranteed or endorsed by the publisher.

## Abbreviations

EGF, Epidermal Growth Factors; HRG, heregulins; HER2, Human Epidermal Growth Factor Receptor 2; NEDD8, Neural Precursor Cell Expressed, Developmentally Down-Regulated 8; IFN, interferon; IL, interleukin; GM-CSF, Granulocyte-macrophage Colony Stimulating Factor; LNMAT1, Lymph Node Metastasis Associated Transcript 1; hnRNPL, Heterogeneous Nuclear Ribonucleoprotein L; MMP9, Matrix Metallopeptidase 9; 5-LOX5-Lipoxygenase; LTB4, Leukotriene B4; PD-1, programmed cell death protein 1; AT1R, Angiotensin II type-1 receptor; TGF, transforming growth factor; TRIF, TIR-domain-containing adaptor inducing interferon-β; uPAR, urokinase-type plasminogen activator receptor; FAK, Focal Adhesion Kinase; PKC, protein kinase C; COX2, Cyclooxygenase 2; PYK2, proline-rich tyrosine kinase 2; TKI, tyrosine kinase inhibitor; ESCC, esophageal squamous cell carcinoma; GC, gastric cancer; TNBC, triple negative breast cancer; HCC, hepatocellular carcinoma; BC, breast cancer; LUAD, lung squamous cell carcinoma; IBC, inflammatory breast cancer; GBC, gallbladder cancer; LLC, Lewis lung carcinoma; LUSC, lung squamous cell carcinoma.
